# VRC01-CLASS PRECURSOR FREQUENCY IN AN AFRICAN POPULATION IS INFLUENCED BY IGHV1-2 ALLELIC COMPOSITION AND NOT MALARIA EXPOSURE

**DOI:** 10.21203/rs.3.rs-9732671/v1

**Published:** 2026-06-24

**Authors:** Michelle K. Muthui, Caleb Kibet, Charity Muriuki, Yiakon Sein, Sharon Owuor, Roselyn Kinoti, Wilfrida Ogonda, Michael Muteti, Oleksandr Kalyuzhniy, John Kimotho, Makobu Kimani, Brenda Kamau, David C. Owuor, Troy Sincomb, Kelly Ramko, Judie Magura, Alfred Muia, Lynn Namwoso, John Orimbo, Christopher A. Cottrell, Omu Anzala, Bernhards Ogutu, Elise Landais, Linda Murungi, Devin Sok, William R. Schief, Thumbi Ndungu, Daniel Muema, Eunice W. Nduati

**Affiliations:** 1 Kenya Medical Research Institute (KEMRI)-Wellcome Trust Research Programme, Kilifi, Kenya; 2 International AIDS Vaccine Initiative (IAVI), Nairobi, Kenya; 3 KAVI –Institute of Clinical Research, University of Nairobi, Nairobi, Kenya; 4 IAVI Neutralizing Antibody Centre, The Scripps Research Institute, La Jolla, California, USA; 5 Department of Immunology and Microbial Science, The Scripps Research Institute, La Jolla, California, USA; 6 Centre for HIV/AIDS Vaccine Development, The Scripps Research Institute, La Jolla, California, USA; 7 Africa Health Research Institute (AHRI), Durban, South Africa; 8 KEMRI Centre for Clinical Research (KEMRI–CCR), Nairobi, Kenya; 9 Ahero Clinical Trials Unit, CREATES/KEMRI–CCR, Kisumu, Kenya; 10 Moderna, Cambridge, MA, USA; 11 HIV Pathogenesis Programme, The Doris Duke Medical Research Institute, University of KwaZulu-Natal, Durban, South Africa; 12 Ragon Institute of Mass General Brigham, Massachusetts Institute of Technology and Harvard University, Cambridge, MA, USA; 13 Institute of Infection, Immunity and Transplantation, University College London, London, UK; 14 Centre for Tropical Medicine and Global Health, Nuffield Department of Clinical Medicine, University of Oxford, Oxford, UK USA

## Abstract

Recent innovations in vaccine design have reignited optimism about the feasibility of developing a safe and effective HIV vaccine. For instance, germline-targeting (GT) immunogens have been designed to selectively expand naïve B cell precursors with broadly neutraliing antibodies (bnAbs)-associated germline-encoded features, thereby priming them for structure-guided maturation. The frequencies and affinities of these precursors in the populations targeted for vaccination are critical to the success of GT vaccine strategies, but limited information is currently available across different geographies and demographics. We set up a study to characterise CD4 binding site-targeting VRC01-class bnAb precursor frequencies in 60 healthy Kenyan adults with varied levels of prior exposure to malaria. Naïve VRC01-class bnAb precursors were detected using the germline-targeting eOD-GT8 immunogen. The overall frequency of these precursors was similar to that reported in North American populations, with IGHV1-2 allelic composition rather than prior long-term exposure to malaria impacting the precursor frequency estimates. Our findings also revealed four new allelic variants of the IGHV1-2 gene that are not currently included in the immunogenetic reference database. Notably, a novel IGHV1-2 allele (IGHV1-2*06_S5416) demonstrated the ability to engage eOD-GT8, unlike the reference *06 allele. Overall, our results support the development of GT vaccines to induce VRC01-class bnAbs in this population and reiterate the need for participant genotyping in GT vaccine trials, particularly in African populations where genetic diversity is high yet understudied.

## INTRODUCTION

A key goal for HIV vaccine development is to elicit broadly neutralizing antibodies (bnAbs) as these would potentially protect individuals from a wide range of infecting HIV clades. Studies in macaques^1–4^, humanised mice^5^ and humans^6^ provide proof-of-principle for bnAb protection against infection with sensitive viruses. However, only a small proportion of HIV-infected individuals naturally generate bnAbs^7–9^. To date, no HIV vaccine candidate has elicited antibodies with sufficient neutralisation breadth and tires by vaccination to prevent infection^10,11^. Consequently, novel reverse vaccinology approaches that utilise insights from structural biology and human immunology^12^, have improved our ability to elicit antibodies of sufficient breadth.

One of these approaches, the germline targeting (GT) approach, seeks to elicit bnAbs by using priming immunogens engineered to bind to diverse precursors for structurally-defined classes of bnAbs, that can then be shepherded towards breadth using successive boosts with immunogens designed to increasingly resemble the native HIV trimer^13–18^. The priming immunogen first needs to overcome the hurdle of activating rare naïve precursor B cells encoding features within their germline B cell receptors (BCRs) that will allow the recognition of epitopes targeted by bnAbs^19^. These precursors will also need to have high affinity for the priming immunogen to outcompete non-precursor B cells^20^. The presence of high affinity bnAb precursors at sufficient frequencies in most individuals across different populations will therefore be important for the success of this approach, and for ensuring that immunogens are relevant for the target population.

The most advanced series of GT immunogens seek to elicit VRC01-class bnAbs that target the envelope CD4-binding site (CD4bs) and share common genetic and structural features^21–23^. These bnAbs are also among the broadest isolated to date^21–24^. VRC01-class bnAbs are defined by their use of immunoglobulin heavy chain variable gene 1-2 (IGHV1-2) of the *02 or *04 alleles^19,25–28^ paired with a light chain (LC) with a short complementarity determining region 3 loop (CDR3) of 5 amino acids (5aa) with a specific motif^29^. VRC01-class bnAb precursor B cells have been characterised in North American populations,^24,27,30^ and were recently described in one study that included African populations^31^ which suggested higher or equivalent frequencies in African populations compared to the US population^31^.

Allelic variation has been shown to impact VRC01-class bnAb precursor frequencies with higher precursor frequencies and VRC01-class responses post eOD-GT8 priming observed in individuals homozygous for IGHV1-2*02^27,28,32^. African populations have high genetic heterogeneity^33,34^ which may impact the allelic composition of their naive repertoires and potentially impact the response to GT immunogens. In addition, individuals in Africa are exposed to a myriad of infectious pathogens that differ from those in Western and other populations, which may modify the naïve B cell repertoire composition^35^, alter immune regulation^36–38^ and/ or modulate vaccine responsiveness as previously suggested^39–44^. Comprehensive knowledge of precursor frequencies and properties, and of factors that may contribute to differences between populations such as environmental exposures, will inform the potential use of germline targeting immunogens in this target population that also bears the greatest burden of HIV.

Malaria is endemic to multiple regions in Africa and has been shown to cause significant immune dysregulation in affected populations^45,46^. The heterogenous levels of exposure to malaria on the continent therefore provide a unique natural experiment to decipher the impact of chronic exposure to a pathogen on the B cell compartment. We thus sought to determine the VRC01-class bnAb precursor frequencies and characteristics in a cohort of individuals living in different geographical regions within Kenya and with varying levels of malaria exposure. In this study we recruited individuals from high, low and intermediate malaria endemic settings. Using the engineered outer domain of gp120 version 8 (eOD-GT8), which targets VRC01-class precursors, as well as other CD4bs bnAb precursors^30,31^, we sorted epitope-specific naïve B cells and performed high-throughput droplet-based single-cell BCR sequencing analysis to identify VRC01-class bnAb precursors, characterise their genetic and functional properties, and estimate their frequencies. We then incorporated participant germline allele inference analysis using IgDiscover^47^ to determine the participants IGHV1-2 genotypes.

We report similar proportions of VRC01-class bnAb precursors between the high and low malaria endemic regions. Additionally, the overall precursor frequency was similar to previously reported estimates from North American populations. We also confirm the association between participant IGHV1-2 genotype and precursor frequency. Importantly, we identified a novel allele (IGHV1-2*06 S5416) with the ability to engage eOD-GT8, unlike the reference *06 allele. These data confirm the potential use of GT immunogens that aim to elicit VRC01-class of bnAbs in this African population.

## RESULTS

### Participant demographic and clinical characteristics:

**Figure 1:**
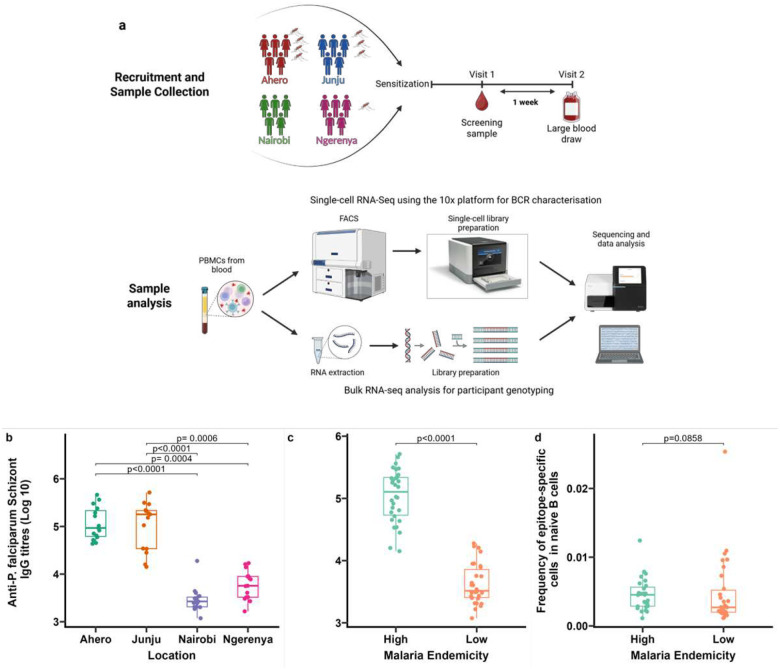
Study design, confirmation of malaria exposure level by *anti-P. falciparum* schizont ELISA and identification of epitope-specific naïve B cells. **a** Schematic showing the regions from which donors were recruited into the study, and the processing of large blood draw samples from samples to identify VRC01-class precursor B cells and genotype participant. **b** Magnitude of *anti P. falciparum* IgG titres per donor stratified by region of recruitment, and **c** by malaria endemicity classification: high – Junju and Ahero, and low – Nairobi and Ngerenya. Statistical significance was determined using the Kruskal-Wallis test with pot hoc analysis done using Dunn’s test with Bonferroni correction applied for multiple comparison adjustment. **d** Frequencies of eOD-GT8-KO11^−^/eOD-GT8^++^ IgD^+^ B cells for each donor stratified by malaria endemicity. Statistical significance was determined using a two-sided Wilcoxon rank sum test. Each dot represents an individual donor, with the thick lines showing the median value, the boxes indicating the 25% and 75% quartiles and the ‘whiskers’ highlighting the minimum and maximum values.

To investigate the potential impact of historical chronic exposure to pathogens on the frequency and characteristics of VRC01-class bnAb precursors, we recruited 60 healthy adults who met the inclusion criteria for collection of a large blood draw, from four regions in Kenya with varied malaria transmission intensities (Fig. 1a and **Supplementary Tables 1 and 2**). The median age of the participants was 26 yrs with a majority being male (68%). Anti-*P. falciparum* schizont IgG antibody levels measured by ELISA showed no difference between Junju and Ahero but were significantly lower in Ngerenya and Nairobi (*p* < 0.0001) (Fig. 1b) confirming that the selection criteria correctly captured individuals with differing levels of historic malaria exposure. Based on these results, participants were grouped into high malaria endemicity (Ahero and Junju) and lower malaria endemicity (Nairobi and Ngerenya) (*p* < 0.0001) for assessment of the impact of malaria exposure on VRC01-class precursor frequencies.

### Identification of CD4bs-specific cells

Fifty-seven of the sixty participants had sufficient PBMCs for antigen-specific cell sorting of bnAb precursors. From these, an average of 30.87 million B cells (interquartile range [IQR]: 18.0 – 50.06 million B cells) were obtained after B cell enrichment per participant (**Supplementary Tables 3 and 4**). Using fluorescence-activated cell sorting (FACS) and the gating strategy illustrated in (**Supplementary Fig. 1**) we defined CD4 binding site (CD4bs) epitope-specific naïve B cells as viable CD3^−^CD14^−^CD16^−^CD56^−^/CD19^+^/IgD^+^/IgG^−^/eOD-GT8-KO11^−^/eOD-GT8^++^ cells. Here, eOD-GT8^++^ denotes binding to eOD-GT8 tetramer probes labelled with two different fluorophores. The eOD-GT8 knockout 11 tetramer probe (eOD-GT8-KO11) contains mutations in the CD4bs that abrogate VRC01-class B cell binding^30,48^. The overall median frequency of epitope-specific cells was 0.0035% (IQR: 0.0022% – 0.0054%) across all donors. There was a trend towards higher frequencies in participants from the high malaria endemicity settings compared to low endemicity settings (0.0045% vs. 0.0027%), although this difference did not reach statistical significance (Fig. 1d, *p* = 0.086).

### The VRC01-class sequences identified show classical signatures of VRC01-class bnAbs

**Figure 2:**
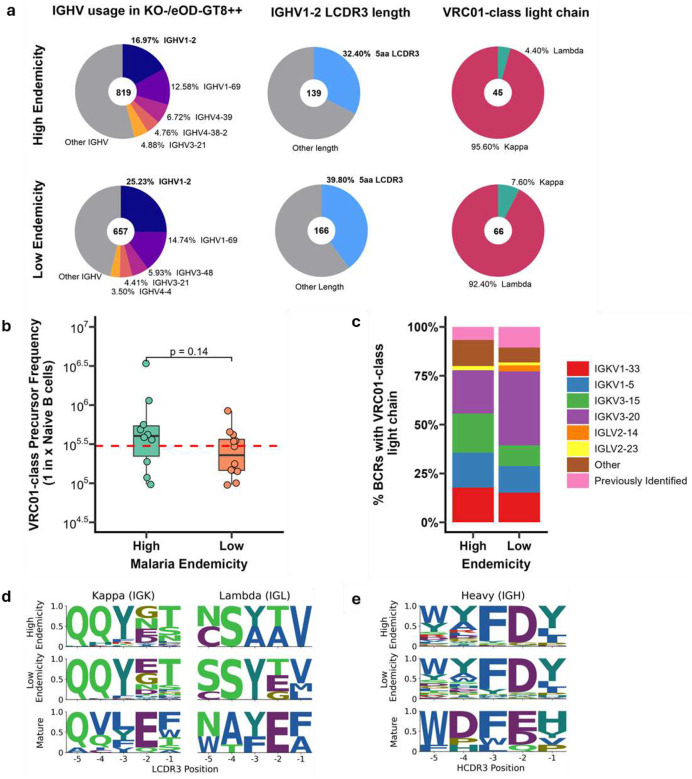
Characteristics of sequences from epitope-specific and VRC01-class naïve B cells. **a** Identification of VRC01-class sequences: first panel – the frequencies of IGHV gene usage among epitope-specific (KO^−^/eOd-GT8^++^) cells. Dark blue shows the frequency of IGHV1-2 gene usage, middle panel – frequency of VRC01-class naïve B cells (IGHV1-2 sequences paired with a 5 aa LCDR3 (light blue)), and last panel – frequency of kappa LC gene use among VRC01-class naive B cells stratified by malaria endemicity setting (high – top row, low – bottom row). **b** Precursor frequency of VRC01-class naïve B cells among total naïve B cells (*n* = 22). Each dot represents an individual donor, with the thick lines showing the median value, the boxes indicating the 25% and 75% quartiles and the ‘whiskers’ highlighting the minimum and maximum values. The red hashed line indicates a precursor frequency of 1 in 300,000 naïve B cells. **c** Percentage of VRC01 class naïve B cells using bnAb κ or λ LC genes, LC genes previously identified in precursors in other studies (pink) or genes not previously identified in precursors, others (brown). **Supplementary Fig. 3b** lists the genes identified in the precursors. **d** Sequence logos of the amino acid sequence of the 5aa LCDR3 of VRC01-class naïve B cells from the high malaria endemicity setting (top row), low endemicity (second row), and mature bnAbs (third row). **e** Sequence logos of the amino acid sequence at the C-terminal end of the HCDR3 of VRC01-class naive B cells highlighting usage at the −5 position (Trp_103– 5_) stratified by malaria endemicity setting (high – top row, low – bottom row).

Using the gating criteria described above, a total of 14,534 epitope-specific cells (eOD-GT8-KO11^−^/eOD-GT8^++^ ) IgD^+^ B cells were sorted (median: 178 cells per participant, interquartile range (IQR) 92 – 321 cells, **Supplementary Table 3**) and subjected to paired B cell receptor (BCR) characterisation to identify VRC01-class signatures. We obtained sequence data from 54/57 donors and proceeded to assess these sequences for VRC01-class sequence signatures. Consistent with previous reports the IGHV1-2 gene was enriched among CD4bs-specific naïve B cells, comprising 16.97% and 25.23% of sequences from the high and low malaria endemicity regions, respectively (overall frequency: 21.1%) compared to its frequency in frequency in general B cell repertoires (4.26% in this cohort with the reported average in literature being 3 – 4%^31,49,50^). IGHV1-2 was the dominant gene family observed in both endemicity settings (Fig. 2a first panel, and **Supplementary Fig 2**). Similar to previous studies, we observed enrichment of 5aa-LCDR3s in LCs paired with a IGHV1-2 HC among the CD4bs-specific sequences in both low (39.8%) and high (32.4%) malaria endemicity settings. Majority of the light chains were derived from the kappa chain and used various kappa and lambda V genes (Fig. 2a middle panel and last panel, and **Supplementary Fig 3a and 3b**).

The VRC01-class signature is minimally characterized by usage of a IGHV1-2*02 or IGHV1-2*04 paired with a 5aa-LCDR3. Using this definition, we detected VRC01-class precursors in approximately 59% of 51 participants with permissive genotypes (30/51) of whom 16 were from high malaria endemicity settings (53%). To minimize unreliable estimates of precursor frequency in samples with very few sequences, we limited the estimation of VRC01-class precursor frequency to 28 participants (52%) that had 10 or more paired HC-LC sequences recovered. Twenty two of the 28 participants had VRC01-class precursors, and for this subset we estimated their VRC01 class precursor frequencies as a proportion of VRC01-class precursors in naïve B cells^31^. Overall, we observed a median precursor frequency of 1 precursor in 345,557 naïve B cells (IQR: 1 in 147,461 – 444,370 naïve B cells). Precursor frequencies were not significantly different between participants from the low compared to the high malaria endemicity settings (median: 1 in 236,200, IQR 1 in 146,092 – 365,896 compared to median: 1 in 402,886, IQR 1 in 231,680 – 542,894 naïve B cells, *p* = 0.14, Fig. 2b). When we compared the overall precursor frequencies from our study with estimates previous studies in North American populations, we found no significant differences in the overall precursor frequencies (*p* = 0.11, **Supplementary Fig c**).

**Figure 3:**
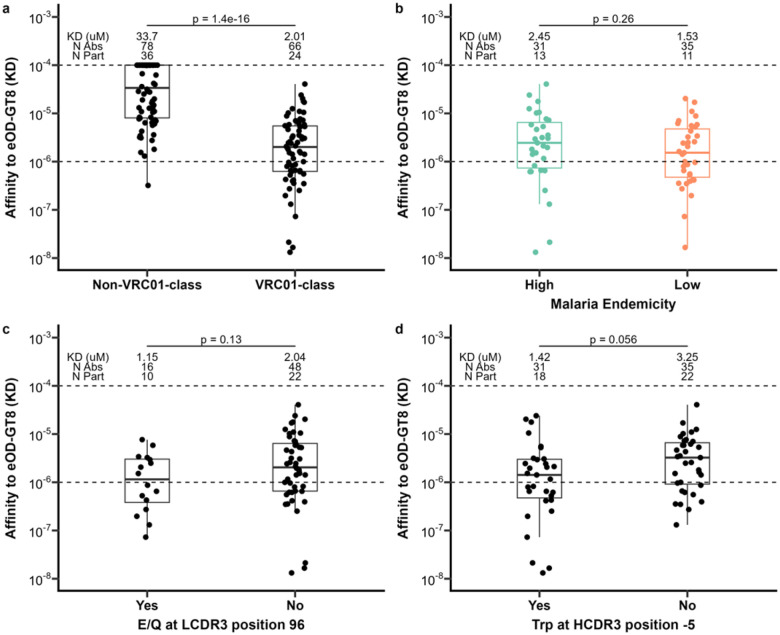
Monovalent affinity of select mAbs produced from VRC01-class and non-VRC01-class sequences derived from epitope-specific cells. **a** Affinity to eOD-GT8 and eOD-GT8 KO11 of mAbs from VRC01-class and non-VRC01-class BCR sequences. **b** Affinity to eOD-GT8 of VRC01-class mAbs from the low and high malaria endemicity settings. **c** Affinities to eOD-GT8 stratified by possession of an E/Q at position 4 and **d** by possession of Trp_103–5_. Each dot represents an individual mAb, with thick lines showing the median value, the boxes indicating the 25% and 75% quartiles and the ‘whiskers’ highlighting the minimum and maximum values. ‘*K*_*D*_ (μM)’ – median *K*_*D*_ in μm, ‘N Part’ – number of participants, ‘N Abs’ – number of mAbs tested. Statistical significance was determined using a two-sided Wilcoxon rank sum test.

In addition to precursor frequencies, we investigated sequence-based metrics associated with VRC01-class BCRs such as light chain V-gene usage, light chain complementarity determining region 3 (LCDR3) motif, and presence of a tryptophan residue at position −5 of the HCDR3 (Trp_103– 5_). Most of the VRC01-class naive B cells (~90%) encoded LC genes used by known VRC01-class bnAbs (Vκ3–20, Vκ1–33, Vκ3–15, Vκ1–5, Vλ2–14 and Vλ2–23) or LC genes previously identified in VRC01-class precursors^27,30^ (Fig. 2c). Approximately 27.1% of the VRC01 class Vκ LCDR3s encoded a glutamate or glutamine (E/Q) at the fourth residue of the 5-aa L-CDR3 (QQYE/QX, Fig. 2d), though none possessed the full QQYEF motif found in mature VRC01 class bnAbs. Interestingly, there was 2-fold enrichment of sequences encoding an E/Q) in sequences from the low endemicity setting (36.4% vs. 17.8%), however this difference did not reach statistical significance (*p* = 0.09, **Supplementary Fig 3d**). For the HCDR3, we observed enrichment of Trp_103– 5_ in 40.8% of VRC01-class precursors (Fig 3d consistent with a previous study^51^, with no differences in enrichment by malaria endemicity (*p* = 0.92, **Supplementary Fig 3e**). Enrichment of J2, that encodes a germline Trp_103– 5,_ was similarly observed (**Supplementary Fig 3f**). The overall HCDR3 length was shorter among the VRC01-class precursors when we compared to control naïve B cells (**Supplementary Fig 3g**).

Noting that many of the key antibody sequence features that mediate the interaction between the CD4bs on gp120 and VRC01-class bnAbs are already encoded in the germline^52^, we generated a ‘bnAb characteristic score’ to assess the number of key features that the isolated VRC01-class precursors share with VRC01-class bnAbs (**Supplementary Data 1**). The bnAb features evaluated were: (i) LCDR3 motif (where each residue was scored based on matching to the QQYEF bnAb motif); (ii) Trp_103–5_ in the HCDR3; (iii) having a short LCDR1 (6 – 7 aa); and (iv) having an HCDR3 length within that of VRC01-class bnAbs (11–18 aa). Out of a maximum possible score of eight, the median score was five, with no significant differences observed between the high and low malaria endemicity settings (**Supplementary Fig 3h**)

Taken together, the results highlight that the VRC01-class sequences identified shared several characteristics with VRC01-class bnAbs. Additionally, there was no significant difference in precursor frequencies by malaria endemicity.

### The VRC01-class sequences identified bind eOD-GT8 with high affinity

We randomly identified 66 paired heavy and light chain sequences from VRC01 class sequences and 78 paired heavy and light chain sequences from non-VRC01 class sequences and expressed them as monoclonal antibodies (mAbs). We then measured their affinity to eOD-GT8 and eOD-GT8 KO11. All the VRC01-class mAbs showed affinity to eOD-GT8 (median *K*_*D*_ *=* 2.01 μM, Fig. 3a), within the range of previously characterized VRC01-class precursors (3.4 μM^24^), with limited affinity to the eOD-GT8-KO11 (median *K*_*D*_ ≥ 100 μM, **Supplementary Fig 4a**). In contrast, only ~58% of the non-VRC01-class sequences showed detectable binding to eOD-GT8, with an approximately 17-fold lower affinity (median *K*_*D*_ ≥ 33.7 μM, *p* < 0.0001). Affinity did not differ between VRC01-class mAbs from the high compared to the low malaria endemicity setting (median K_*Ds*_ = 2.54 μM and 1.53 μM respectively, p = 0.256, Fig. 3b). We observed a trend towards higher affinity in monoclonal antibodies from sequences with an E/Q at position 96 (~1.8-fold, Fig. 3c) and those encoding a Trp_103–5_ (~2.3-fold, Fig 3d), however this was of borderline significance for Trp_103–5_ (*p* = 0.0564) and not significant for E/Q at position 96.

Light chain gene usage has previously been used to define VRC01 sub-classes, namely VRC01 (Vκ3–20+), VRC23 (Vκ3–15+), N6 (Vκ1–33+), PCIN63 (Vκ1–5+) and VRC-PG20 (Vλ2–14)^30^. We did not observe any significant differences in median affinity between the subclasses, however, of the three highest affinity mAbs, two were from the VRC23 subclass and one from the N6 sub-class (median *K*_*D*_ = 21.3 nM, 16.6 nM for VRC23 and 13.2 nM for N6, **Supplementary Fig. 4b**). The N6-subclass of bnAbs is known for its potency and breadth^53^, and along with VRC23 does not require LCDR1 deletions to acquire breadth^30,54^. Finally, we evaluated whether the bnAb score associated with precursor affinity and observed a modest positive correlation between the bnAb characteristic score and affinity to eOD-GT8 (ρ = −0.35, *p* = 0.0047, **Supplementary Fig. 4c**).

In summary, the identified VRC01-class precursors showed strong affinity to eOD-GT8, with a non-significant trend toward higher affinity observed in mAbs encoding a Trp_103–5_ in the HCDR3. Additionally, the more features the precursors shared with the VRC01-class bnAbs, the better the affinity to eOD-GT8 was.

### The IGHV1-2*02 allele was associated with higher VRC01-class precursor frequencies

**Figure 4:**
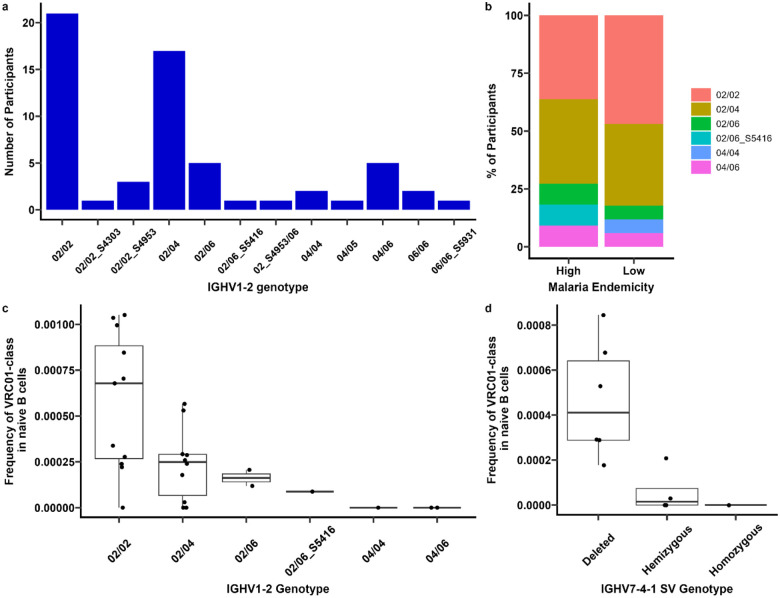
IGHV1-2 genotype distribution and impact on VRC01-class precursor frequency. **a** Distribution of identified genotypes for all donors in the study (N = 60). **b** Distribution of identified genotypes for donors included in the assessment of precursor frequencies (*n* = 28). Novel alleles that encoded the same amino acid sequence as the reference allele were reassigned as the reference allele. **c** Boxplots showing the frequency of VRC01-class naïve B cells for each stratified by IGHV1-2 genotype (*n* = 28). **d** Frequency of VRC01-class naïve B cells stratified by IGHV7-4-1 genotype (*n* = 11). Each dot represents an individual mAb, with thick lines showing the median value, the boxes indicating the 25% and 75% quartiles and the ‘whiskers’ highlighting the minimum and maximum values.

Given the reported influence of IGHV1-2 genotype on IGHV1-2 gene usage^27^, we determined the IGHV1-2 genotypes for all 60 study participants. Twelve different IGHV1-2 genotypes were identified consisting of combinations of *02, *02_S4303, *02_S4953, *04, *05, *06, *06_S5416 and *06_S5931 alleles (Fig. 4a). Alleles *02_S4303, *02_S4953, *06_S5416 and *06_S5931 identified in 1, 4, 1 and 1 participants respectively, represent novel alleles. IGHV1-2*02_S4953 allele has previously been identified in Caucasian population^28,32^ and its existence validated in genomic DNA^55^. Similarly, *06_S5416 and *06_S5931 have been identified in repertoire studies done in other populations^56,57^.

The *02_S4303 and *02_S4953 alleles encoded the same amino acid sequences as the reference *02 allele and hence we classified them as *02 in subsequent analyses (**Supplementary Fig. 5**). On the other hand, the *06_S5416 and *06_S5931 alleles encoded non-synonymous nucleotide changes hence they were considered distinct from the reference *06 allele. The *06_S5931 allele encodes a valine to methionine mutation at position 11 (V11M), a mutation acquired by some HIV bnAb lineages (e.g., N6 and DH270) during maturation^58^. As expected, we did not detect any precursors from this allele due to the lack of the W50 residue. Overall, *02/*02 was the most dominant genotype identified followed by *02/*04, *02/*06, *04/*06, *06/*06, *04/*04, and *04/*05 in order of decreasing frequencies, with only 5% (3/60) of participants lacking either a *02 or *04 allele. The genotype distribution did not differ between participants from low and high endemicity regions (Fig. 4b).

The highest precursor frequencies were associated with the IGHV1-2*02 allele, with participants homozygous for the *02 allele having higher VRC01-class precursor frequencies (~2.7-fold higher than *02/*04 participants and ~4.2-fold higher than *02/*06 participants, Fig. 4c), consistent with its association with higher IGHV1-2 gene usage (**Supplementary Fig. 6a**). Homozygous loss of expression of the IGHV7-4-1 gene has also been shown to influence IGHV1-2 gene usage levels, with individuals homozygous for the deletion showing ~2.8-fold higher gene use than those with both copies of the gene present^55^. We inferred IGHV7-4-1 deletion in 27 participants who were heterozygous for the IGHJ6 gene (**Supplementary Table 6**) and observed a 1.8-fold increase in IGHV1-2 gene use in participants with IGHV7-4-1 gene deleted compared to those with both copies of the gene present, though the difference did not reach statistical significance (*p* = 0.082, **Supplementary Fig. 6b**). Higher VRC01 precursor frequencies were also observed in individuals inferred to lack the IGHV7-4-1 gene, although sample sizes in each genotype group were limited (Fig. 4d).

In summary, we observed several novel IGHV1-2 alleles among the 60 participants, highlighting the genetic diversity present within this population, as well as a strong influence of IGHV1-2 genotype on precursor frequencies.

### A novel IGHV1-2*06 allele may have ability to engage eOD-GT8

The IGHV1-2*06_S5416 allele encoded a Glutamine (Q) instead of the canonical Arginine (R) residue at aa position 50 (Kabat numbering). This mutation is located at the start position of the conserved ‘WNR’ VRC01-class motif that is important for engaging eOD-GT8^19,59^. Interestingly, we identified one potential VRC01-class precursor that derived from the 06_S5416 allele suggesting that the R50Q mutation may have conferred some ability to engage with eOD-GT8. We produced a mAb from the BCR sequence of this allele and indeed, the mAb showed detectable binding to eOD-GT8 with a *K*_*D*_ of 2.95 μM with no binding to eOD-GT8 KO11 (*K*_*D*_ of 100 μM). This was similar to the median *K*_*D*_ observed for VRC01-class precursors deriving from *02 and *04 alleles (median *K*_*D*_ 2.01 μM, Fig. 3a). Nevertheless, the other eight precursors identified from this individual were derived from the *02 allele potentially implying bias against the use of the *06_S5416 allele. This finding suggests that there could be other novel IGHV1-2 alleles in existence with important implications for eOD-GT8 binding.

### Broad range of CD4bs lineage precursors identified, with the non-VRC01-class VH1-2 naïve B cell repertoire showing some conservation including a dominant shared public light chain clonotype.

**Figure 5:**
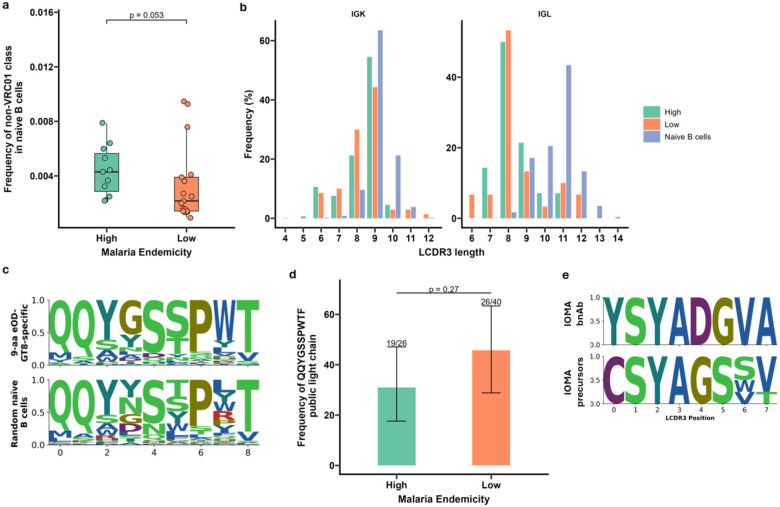
Sequence characteristics of non-VRC01-class eOD-GT8-specific naïve B cells. **a** Frequency of non-VRC01-class epitope-specific cells in naïve B cells (participants with ≥10 sequences, *n* = 28). **b** LCDR3 length of non-VRC01-class VH1-2^+^ CD4bs-specific naïve B cells. **b** Amino acid motif of non-VRC01-class VH1-2^+^ CD4bs-specific naive B cells with a 9-aa LCDR3 and control B cells with a 9aa-LCDR3. **c** Frequency of the public LC in sequences from the low and high malaria endemicity settings. **d** Amino acid motif of IOMA bnAb (top row) and the IOMA-class naïve precursors (bottom row, *n* = 7).

In addition to engaging VRC01-class precursors, eOD-GT8 also engages other CD4bs-targeting bnAb precursors^30,31^. Majority of the non-VRC01-class repertoire also shows specific binding to eOD-GT8 (Fig. 3a and **Supplementary Fig. 4a**) though with overall lower affinity compared to VRC01-class bnAbs. Interestingly, we did observe a trend towards a higher frequency of non-VRC01-class naïve B cells in the sequences from the high malaria endemicity setting (*p* = 0.053) (Fig 5a). though there were no differences in the non-VRC01-class naïve B cell affinity by malaria endemicity (*p* = 0.37, **Supplementary Fig. 7a**).

Aside from IGHV1-2+ B cells paired with a 5-aa L-CDR3 (VRC01-class), we identified several other eOD-GT8^++^/KO^−^ VH1-2+ naive B cells. Though there was some enrichment for shorter LCDR3 lengths (6, 7 and 8 aa) in the Vκ light chains when compared to control naïve B cells (Fig. 5b), the most frequent LCDR3 length observed was 9 aa (57.1% and 45.6% in the high and low malaria transmission settings, respectively). Among these epitope-specific 9-aa VH1-2^+^ B cells, we observed an enriched LCDR3 motif ‘QQYGSSPWTF’ associated with IGκ3–20 + Jκ1–01 light chains (Fig. 5c and **Supplementary Fig 7b**) that was present in multiple individuals (**Supplementary Fig 7c**). This public light chain was previously identified in 33% of VH1-2^+^ HCs paired to a 9-aa L-CDR3^30^, and was present in our study at a frequency of 37.6% with no difference in occurrence by malaria endemicity (Fig. 5d). We also observed other public light chains that were present in multiple individuals, however these were less frequent (< 8%, **Supplementary Data 3**).

Similar to Vκ light chains, short LCDR3s were also enriched in the Vλ light chains, particularly lengths of 6, 7 and 8 aa (Fig. 5b). We identified seven eOD-GT8-specific naïve B cells with an IOMA signature, VH1-2^+^ paired with an 8-aa λ2–23 LCDR3. All the IOMA-class sequences matched positions 2, 3 and 4 of the mature IOMA bnAb LCDR3 (Fig. 5e). Other non-VRC01 class CD4bs bnAbs use diverse HC and LC combinations to engage eOD-GT8, this can occur via HCDR3 loop-dependent binding (e.g., CH103, VRC16 and VRC13 class bnAbs) or by using their HCDR2 region to mimic CD4 (VH1–46 derived bnAbs e.g., 8ANC113)^60^. Within the non-VRC01 class CD4bs naïve B cells, we observed several sequences with signatures similar to CH103, VRC13 and VRC33 CD4bs bnAbs as well as several VH1–46+ B cells (**Supplementary Table 5**). The non-VRC01 class CD4-bs repertoire thus represents additional B cell lineages that can be engaged for maturation into CD4bs-directed bnAbs.

## DISCUSSION

Germline-targeting is a promising strategy to induce HIV bnAbs by vaccination. Four proof-of-principle trials conducted so far (IAVI G001^28^, IAVI G002^61^, IAVI G003^61^, and IAVI C101^23^) have provided evidence that efficient priming of VRC01-class responses using a GT immunogen can be achieved in North American and African populations. The G002 trial additionally provided preliminary evidence that primed VRC01-class bnAb precursors can be shepherded towards bnAb development. These results are encouraging for HIV vaccine development efforts, particularly for African populations where the HIV burden is highest. The study described here sought to isolate and characterise VRC01-class bnAb precursors in healthy Kenyan adults experiencing varied levels of malaria transmission to assess the potential impact of chronic malaria exposure on VRC01-class naïve bnAb precursor frequencies.

We identified VRC01-class naïve B cell precursors in 59% of the participants evaluated, though it is possible that the remaining participants also had VRC01-class precursors, but insufficient cell numbers and/or low precursor frequency may have hindered identification. In the population studied here, precursor frequencies did not differ significantly from previous studies in North American populations, with the overall precursor frequency estimate (1 precursor in 345,557 naïve B cells) being similar to previous estimates (1 precursor in 300,000 naïve B cells^24,30^). We postulated that chronic life-long exposure to malaria may drive alterations in the naïve B cell compartment, potentially impacting the HIV CD4bs-specific antibody repertoire. A previous study showed some differences in IGHV gene usage within classical memory and atypical memory B cells subsets driven by malaria exposure^62^. The authors did not observe malaria-related changes in the naïve B cell compartment; however, the sample size of malaria-infected participants included in the analysis was quite limited (three participants).

Infection-related immune impairment has also been observed following measles infection where restructuring of the naïve B cell compartment led to a decrease in naïve B cell diversity in a cohort of unvaccinated children, with alterations in IGHV-J gene usage frequencies observed in 10% of the cohort that persisted post measles resolution^35^. Contrary to our hypothesis, naïve VRC01-class bnAb precursor frequencies were not influenced by prior chronic exposure to malaria, which is reassuring for future vaccine implementation efforts in endemic populations. There remains a possibility however that chronic exposure to other pathogens may impact precursor frequences or that chronic malaria exposure may have impacted other repertoire subsets important for responses to other pathogens, therefore this is still an area that requires investigation.

Consistent with other studies, precursor frequencies were influenced by the IGHV1-2 genotype^27,32,59^. Individuals homozygous for the *02 allele had the highest median precursor frequency estimates compared to all other permissive genotypes identified in our study (*02/*04, *02/*06, *04/*04 and *04/*06). The higher precursor frequency associated with the *02 allele also correlated with the level of VRC01-class response observed post eOD-GT8 vaccination in the IAVI G001 trial^32^. Encouragingly, ~82% of the study participants had an *02 allele, with only five percent (3/60) lacking an *02 or *04 allele, supporting the development of GT vaccines to induce VRC01-class bnAbs in this population. Notably, IGHV1-2 genotype did not explain all the variation in precursor frequency estimates in our study, nor did it explain all the variation in post-vaccination VRC01-class responses in G001, suggesting a role for other factors such as immune history or additional genetic factors^32^.

Indeed, other genetic loci are also proposed to influence IGHV1-2 gene use, for example homozygous deletion of the IGHV7-4-1 gene that has been associated with higher gene use^55^. Our observations are in line with this, and we similarly observed a trend towards higher VRC01-class precursor frequencies in individuals lacking expression of the IGHV7-4-1 gene. We do acknowledge, however, that the small sample size of participants with overlapping haplotyping and VRC01-class precursor frequency data limited our ability to perform more comprehensive analyses. We also acknowledge the fact that inference of germline repertoire deletions from RepSeq data may not be efficient for poorly expressed alleles such as IGHV7-4-1*01. Further studies on the possible impact of the IGHV7-4-1 gene deletion on VRC01-class precursor frequencies and vaccine responses and how this compares to/or interacts with the impact of IGHV1-2 genotype are warranted.

In addition to precursor frequencies, precursor affinity is also an important consideration for the success of GT immunogens^20,63,64^. IGHV1-2 genotype has an impact on precursor affinity, with the *05 and *06 unable to bind eOD-GT8 due to a lack of a tryptophan residue at the start of the conserved ‘WNR’ motif critical for engaging eOD-GT8^19,27,32^. Strikingly, we identified a novel *06-like allele (IGHV1-2*06_S5416) encoding a non-synonymous R50Q mutation that we postulate may have conferred some ability to engage with eOD-GT8, however this finding warrants confirmation. The potential functional consequences of novel alleles within the IGHV1-2 genes, and the potential for some of these mutations to be in SHM regions important for bnAb development reiterates the importance of genotyping participants in GT clinical trials.

Aside from IGHV1-2 genotype, the presence of Trp_103–5_ showed evidence of an association with increased affinity of VRC01-class mAbs to eOD-GT8. Trp_103–5_ forms hydrogen bonds with Loop D of gp120^29^, and antibodies encoding this key residue may more readily develop into bnAbs as they require fewer maturation steps^59^. In addition to Trp_103–5_ other sequence features required for VRC01-class bnAb development, such as use of VH1-2*02 or *04 allele usage and the presence of a 5-aa LCDR3 can readily be encoded in the germline^52^ hence it is likely that the more of these features a naive VRC01-class precursor possesses, the more likely it is to be shepherded towards VRC01-class bnAb development by vaccination. By scoring BCR sequences on the number of bnAb-like characteristics they possessed, we found that a higher score correlated with better affinity to eOD-GT8. In future, as full regimens develop, it will be interesting to evaluate whether the bnAb score of VRC01-class bnAb precursors correlates with accelerated maturation towards broad neutralisation.

In conclusion, we confirm that VRC01-class naïve B cell precursors are present in Kenyan populations, and that the precursor frequency does not seem to differ from that observed in North American populations, nor to be impacted by chronic exposure to malaria. We note that several of these precursors share critical sequence characteristics with VRC01-class bnAbs and hence may be readily shepherded towards breadth by a suitable vaccination regimen. We also identified four novel IGHV1-2 alleles, one that may have functional consequences for binding to eOD-GT8. These data underscore the value of participant IGHV genotyping in GT trials and suggest that rare, function-restoring variants can exist. Moreover, GT immunogens to induce bnAb responses to other target sites on the HIV virus envelope are in development, hence it remains necessary to continue to identify allelic variants that may modulate vaccine responses for other bnAb classes in diverse populations.

## METHODS

### Study design and participant recruitment

Ethical approval for participation in these cohort studies was given by the Kenya Medical Research Institute Scientific and Ethical Review Unit (KEMRI/SERU/4177). Participants were recruited from four regions in Kenya with varying malaria endemicities. These included Ahero in Western Kenya where there is high malaria transmission, Nairobi (central Kenya) where there is low to no malaria transmission and from two regions in Coastal Kenya, Junju with moderate transmission and Ngerenya with low transmission intensity. Since historical exposure to malaria could vary among Nairobi residents based on previous travels out of the city to malaria-endemic ancestral locations, inclusion criteria in Nairobi included pre-screening to identify individuals who had no to low detectable levels of anti-*P. falciparum* schizont antibodies as a proxy measure of no to low historical malaria exposure (anti-*P. falciparum* schizont ELISA described elsewhere^65^). Adults between the ages of 18 – 60 years were considered for recruitment if they had lived in the recruitment area for at least the last 10 years. A screening visit was first carried out where adults from each site were invited to the study health facilities for examination. A five ml blood sample was taken for HIV and malaria infection screening, haemoglobin level assessment and complete blood counts. Additionally, medical history was also taken to determine eligibility. Detailed inclusion and exclusion criteria are provided in **Supplementary Table 2**. Following the screening visit, 15 participants from each study site who were deemed fit to provide one blood bag (450 ml blood draw) were recruited into the study. The blood draw was conducted within one week of screening.

### Sample collection and PBMC processing

Whole blood from the blood bags was layered onto density centrifugation media (Histopaque, Sigma Aldrich^™^, catalogue number (#)10771) (15 – 27 mL of blood to 10 mL of Histopaque) and centrifuged at 400 x *g* for 40 minutes. The PBMCs were then harvested and washed once using Hanks balanced salt solution (HBSS, Sigma Aldrich^™^, #H8264) and then a second time using RPMI (Sigma Aldrich^™^, #R0883) 10% HIFCS (Sigma Aldrich^™^, #F4135) medium (R10) before cell counts were determined on a Vi-Cell XR automated cell counter. The PBMCs were then resuspended in cold freezing media (10% DMSO (Sigma Aldrich^™^, #D2650) in HIFCS) at a concentration of 1.5×10^7^ or 2.0×10^7^ cells/mL, and 1 mL of cell suspension transferred to pre-labelled cold cryovials. The cryovials were then transferred to a pre-chilled Mr. Frosty^™^ Freezing Container (Thermo Scientific^™^, #5100–0001), and the container placed in a −80°C freezer overnight. The next day the frozen PBMCs were transferred to liquid nitrogen tanks for long term storage.

### Sort probe preparation

Sort probes used to isolate epitope-specific cells were prepared as previously described^27^. Briefly, biotinylated eOD-GT8 wildtype (WT) and knock-out (KO) monomers were combined with fluorescently labelled streptavidin (SA-AlexaFluor647 (Invitrogen^™^, #S21374) and SA-AlexaFluor488 (Invitrogen^™^, #S11223) for the WT baits and SA-Brilliant Violet 421 (BioLegend^™^, #405225) for the KO bait) at a 4:1 molar ratio. The performance of the probes was then tested using beads coated with monoclonal antibodies of known binding characteristics to the probes (germline-VRC01 or KG064–099, for testing eOD-GT8 WT and eOD-GT8-KO probes respectively).

### B cell enrichment, flowcytometry and epitope-specific B cell sorting

Cryopreserved PBMC samples were thawed and resuspended in pre-warmed R50 media (RPMI-1640 containing 50% HIFCS). The PBMCs were then pelleted by centrifugation at 500 x g for 7 minutes, supernatants decanted and resuspended in FACS buffer (2% HIFCS, 25 mM HEPES (Gibco^™^, #H0887) buffer, 2 mM EDTA (Sigma Aldrich^™^, #03690) and 1 x PBS (Gibco^™^, #10010–015). The PBMC were counted and the concentrations adjusted to 5 × 10^7^ cells/mL for B cell enrichment using the EasySep^™^ Human Pan-B Cell Enrichment Kit (STEMCELL^™^ Technologies, #19054) according to manufacturer’s instruction. The enriched B cells were counted and the quantities of antibody to use for staining determined (for every 10 – 20 million B cells we used a final staining volume of 100 μL made up of 5 μL of fluorescently-labelled cell phenotyping markers (**Supplementary Table 6**), 2 μL of sample multiplexing hashtag (BioLegend TotalSeqC) and 10 μL of the probes). The enriched B cells were stained using the antibody and KO probe (100 nM final staining volume) cocktail for 15-minutes incubation on ice and in the dark. The WT probes were then added (100 nM) to the B cells and incubated for a further 30 minutes. Zombie Aqua^™^ amine-reactive viability dye (BioLegend^™^, #423102) was then prepared at dilution of 1:300 in FACS buffer (1 mL for every 10 – 20 million B cells) and added to the cells, followed by incubation for 30 minutes in the dark. The cells were then washed with 10 mL FACS buffer and resuspended at a concentration of 15,000 cells/μL in FACS buffer for acquisition on a FACS Melody, using the gating and sorting strategy illustrated in **Supplementary Fig 1**. Epitope-specific naïve B cells were defined as IgD+ B cells that bound both eOD-GT8*AF647 and eOD-GT8*AF488 (‘double-positive’) but did not bind the eOD-GT8-KO*BV421 probe. Epitope-specific cells were sorted into 20 μL of 100% FBS. We also sorted between 700 to 1500 random naïve B cells from each donor into the same well as the epitope-specific cells to increase the total cell numbers to above 500 which is the recommended minimum number of cells that can be profiled using the 10x NextGEM reagents. The random naïve B cells were separately hashtagged and to allow removal from the sequence dataset before analysis. After the sort, the sorted epitope-specific cells were resuspended in 200 μL of cold PBS and pelleted by centrifugation at 2500 rpm for 2 minutes. Excess PBS was then aspirated to leave 38.7 μL volume required for Gel-beads-in emulsion (GEMs) preparation using the 10x Genomics^®^ platform.

### Preparation of single-cell RNAseq libraries using the10x genomics platform

Single-cell RNAseq libraries of the epitope-specific B cells were prepared using the Chromium Next GEM Single Cell 5’ Reagent Kit v2 with Feature Barcoding technology according to the manufacturer’s instructions. Immunoglobulin genes were amplified from the fraction of cDNA prepared from cellular mRNA and used to prepare V(D)J libraries, while hashtag libraries were prepared from the cell-hashtag oligonucleotide-derived cDNA. Quantification of the libraries was done by qPCR using the KAPA Library Quantification Kit for Illumina^®^ platforms (Roche^™^, #KK4824) and library sizes determined using the Agilent Bioanalyzer platform. Libraries were then pooled to achieve a minimum of 5000 reads per cell for both the V(D)J libraries and cell hashtag libraries. The pooled libraries were sequenced on an Illumina NextSeq500 using a 150-cycle high output kit with read lengths set to 26 base pairs (bp) for read 1, 10 bp for index 1, 10 bp for index 2 and 90 bp for read 2.

### Next generation sequencing (NGS) library preparation for IGHV1-2 genotype analysis

Bulk RNAseq libraries for genotyping using IgDiscover were prepared using primers from Bernat et al., 2019^66^. Briefly, frozen PBMCs from each study participant (N = 60) were thawed and B cells enriched as described above. The enriched B cells were pelleted by centrifugation at 400 x g for 5 minutes, and the pellets resuspended in TRIzol reagent (Invitrogen^™^, #10296010) at a ratio of 1 mL TRIzol to 100 uL of B cell pellet. Two hundred microlitres of chloroform were added to the TRIzol sample before incubating for three minutes at room temperature after mixing vigorously. The sample was then centrifuged at 1400 x g for 35 minutes at 4°C. The aqueous phase was then harvested and transferred to a 1.5 mL microcentrifuge tube containing 2 μL of GlycoBlue^™^ (Invitrogen^™^, #AM9515) and 500 μL of isopropanol (Sigma Aldrich^™^, #I9516), mixed and incubated overnight at 4°C.

The next day, the sample was spun at 13,000 rpm for 30 minutes at RT, and the pellet then washed gently using 1 ml of ice-cold 75% ethanol and pelleted by centrifugation for 5 min at 7500 x g at 4 degrees two times. The ethanol was decanted and the pellets air-dried for no longer than 5 minutes. Forty microlitres of RNase-free water was then added to each sample and the RNA dissolved by heating the mix at 60°C for 10 minutes. From the extracted RNA, cDNA was prepared using an IgM primer containing a unique molecular identifier (UMI) and the Illumina Read2 sequence. Five prime multiplexed libraries were then prepared using IGHV leader region-specific primers and a universal reverse primer. After this, the 5 ng of each library was then individually indexed. A pool of 15 samples was prepared at equimolar ratios then sequenced on an Illumina MiSeq (version 3 kit, 2 × 300 cycles, #15043762) at 13 pM with 15% PhiX (Illumina, #15017872) spike.

### Production of monoclonal antibodies and SPR affinity measurements

MAb production was outsourced to Genscript where the monoclonal antibodies were produced as human IgG1 using the TurboCHO expression service. We measured kinetics and affinity of antibody-antigen interactions on a Carterra LSA using a HC30M sensor chip that has polycarboxylate hydrogel, medium charge density, 30nm coating thickness, moderate ligand capacity and excellent diffusion characteristics for monomeric antigens. We used 1x HBS-EP+ supplemented with BSA at 1mg/ml at pH 7.4 as running buffer. Using manufacturer’s instructions about 2800–3300 RU of capture antibody was amine coupled using the N-Hydroxysuccinimide (NHS) and 1-Ethyl-3-(3-dimethylaminopropyl) carbodiimidehydrochloride (EDC) Amine Coupling Kit using 10 times diluted NHS and EDC for final concentrations of NHS and EDC at 1.15 mg/ml and 7.5 mg/ml respectively. The chip surface contact time of EDC/NHS solution varied between 1 and 3 minutes. The Southern Biotech capture antibody was used at a concentration of 25–100 μg/ml with 10 minutes contact time. We used 1.7% phosphoric Acid as the regeneration solution with 60 seconds contact time and injected three times per each cycle, with ligand concentration maintained at approximately 1 μg/ml with 5 min contact time. Analyte concentrations were quantified on NanoDrop 2000c Spectrophotometer using absorption signal at 280 nm. Raw sensograms were analyzed using the Carterra Kinetics software. Where a measurement did not result in kinetic-fit *K*_*D*_ from the Carterra Kinetics software analysis, the *K*_*D*_ value was set to ≥100 μM.

### BCR Sequence processing for downstream analysis

#### Analysis of immunoglobulin genes of epitope-specific (KO-/eOD-GT8++) naive B cells

A standardized “*immbase”* analytical pipeline was developed by modifying the “G00X” pipeline previously used for analysis of the IAVI-G00X clinical trials^28^. The modified pipeline uses CellRanger version 7.1.2 with *cellranger multi* commands, instead of the *count* and *vdj* commands used by the “G00X” pipeline and performs detailed analyses of the sequence features for both VRC01-class and non-VRC01-class BCRs (**Suppl. Fig 8**). The “*immbase*” pipeline consists of five main steps: demultiplexing, converting BAM files to FASTQ format, V(D)J processing, Adaptive Immune Receptor Repertoire (AIRR) output generation, and report generation. For AIRR output, the pipeline performs B cell receptor (BCR) V gene assignments, heavy and light chain pairing and somatic hypermutation (SHM) analysis, using the Sequencing Analysis and Data Library for Immunoinformatics Exploration (SADIE, https://github.com/jwillis0720/sadie).

A manifest file was created to automate the analysis pipeline and statistical calculations. This manifest captured the GEM number, sample ID, hashtag or cell multiplexing oligo (CMO) ID, number of cells sorted, number of cells in the GEM, Sample index, FASTQ ID, run number, path to FASTQ files, sample type, and endemicity. The sequences were first demultiplexed to separate the different library types (either V(D)J and feature barcode (FBC) libraries) as well as individual samples that were pooled in the same GEM reaction. Next V(D)J annotation was carried out and the pairing of heavy and light chains determined by extracting productive sequences and categorizing them based on their loci (IGH, IGK, IGL). We included only paired HC + LC sequences and filtered the B cell isotype to IgM and/or IgD B cells since we had sorted na ve B cells. To exclude potential IgM memory cells, that as expected, would show additional mutation, we also included an additional filter. A per-sequence mutation cut-off of below 2% was used to exclude sequences with potential SHM while allowing retention of sequences derived from novel alleles absent from the reference datasets used for the annotation.

Next, we identified VRC01-class features based on the described sequence characteristics: usage of the IGHV1-2*02 or IGHV1-2*04 alleles and a light chain CDR3 length of 5 aa. Additionally, the pipeline computed sequence-level statistics, including the number of sequences per sample, the frequency of VRC01-class and IOMA-class sequences. IGHV1-2 gene usage patterns were assessed by calculating the relative frequency of V-gene segment utilization across samples. To classify potential CD4bs antibody lineages, we utilized predefined categories based on their sequence characteristics (**Supplementary Table 5**).

We used the formula below to estimate the VRC01-class precursor frequency in na ve B cells. Estimates of the frequencies of VRC01-class naïve precursors were limited to participants with greater than 10 sequences recovered to minimize the impact of low sampling depth on the frequency estimates.


$$\frac{\text{Number}\;\text{of}\;\text{IgD}+\text{epitope}-\text{specific}\;\text{cells}}{\text{Number}\;\text{of}\;\text{IgD}+B\;\text{cells}}\times \frac{\text{Number}\;\text{of}\;VRC01-\text{class}\;\text{sequences}}{\text{Number}\;\text{of}\;\text{sequenced}\;\text{epitope}-\text{specific}\;\text{cells}}\times 100\%$$

#### Dinition of IGHV1-2 genotypes and VH1-2 gene usage frequency

Sequence reads from each bulk RNAseq library generated for each donor were processed using the IgDiscover analysis pipeline (version 1.0.2.dev4+g7975573) for IGHV germline inference using the default parameters. The number of merged reads for all 60 participants are provided in **Suppl. Data 2** (median reads: 1,198,028, IQR 881,304 – 1,413,149 reads). To confirm the genotyping and identified novel IGHV1-2 novel alleles, the libraries were also genotyped using the corecount genotyping module in IgDiscover^67^, with germline gene inference and genotyping also analysed using TIgGER analysis tool (version 1.1.2)^68^. We observed good concordance between the two analysis methods (**Suppl. Data 2**). IGHV1-2 gene usage summaries were generated using the ‘countGenes’ function within the Alakazam package (version 1.4.2)^69^.

#### Haplotype analysis to determine IGHV7-4-1 deletion

We used haplotype-based inference to assess IGHV7-4-1 deletion in a subset of 28 participants (**Supplementary Data 2**) who were heterozygous for IGHJ6 (expressed both IGHJ6*02 and IGHJ6*03 alleles), a commonly used ‘anchor gene’ to define the chromosome from which expressed IGHV genes were derived. As recommended, following novel allele discovery, we reassigned alleles using an individual’s personal genotype then filtered the dataset to retain sequences with no more than three mutations called in the V gene and no mutations in the D gene (mutation analysis was done using SHazaM^69^ analysis tool, version 1.3.1). After this, we collapsed the sequences by clonotype to retain only unique sequences. We then employed RAbHIT tool (version 0.3.0),^70^ that adapts a Bayesian framework to infer V, D or J haplotypes, to infer zygosity using the default parameters, however, we set the minimum minor allele fraction of the anchor gene to 0.275 as opposed to 0.3. To detect genes deleted from either or both chromosomes, we first used the ‘deletionsByBinom’ function to detect genes deleted from both chromosomes. We then carried out haplotype inference for the non-deleted genes using the ‘createFullHaplotype’ function to infer single-chromosome deletion events. The priors for Bayesian inference were inferred from the usage frequencies of the anchor gene (IGHJ6). Where IGHV7-4-1 gene deletion was defined as ‘unknown’ for a sample, we excluded that sample from assessment of impact on IGHV1-2 gene usage. We obtained data from 27 of the 28 participants, with only one donor’s IGHV7-4-1 deletion status defined as unknown.

### Statistical analysis

Participant demographic characteristics and summaries of B cell sorting and sequencing metrics were stratified by location and malaria endemicity and reported as medians with interquartile ranges (IQR) or summarised as counts and percentages. Comparisons between two independent groups were performed using the Wilcoxon rank-sum test for continuous data and the Chi-Square test for comparisons of frequencies of categorical variables with 95% confidence intervals calculated using the Clopper-Pearson method for binomial proportions. For comparisons of more than two independent groups, we used the Kruskal-Wallis test with post-hoc analysis done using Dunn’s test with the Bonferroni correction applied to adjust for multiple comparisons. We tested correlations between two continuous variables for significance using the Spearman’s test. Statistical and graphical analyses were performed using R (version 4.4.3) using the following packages: tidyverse (version 2.0.0), ggprism (version 1.0.7), ggpubr (version 0.6.3), ggsignif (version 0.6.4), stringr (version 1.6.0), RColorBrewer (version 1.1–3), arrow (version 23.0.1.1), rstatix (version 0.7.3) and DescTools (version 0.99.60). Sequence logos for VRC01-class antibodies were visualized using the Python-based Logomaker^71^ and pie charts drawn using Prism (GraphPad, version 10.3.1).

## Supplementary Files

This is a list of supplementary files associated with this preprint. Click to download.

- SupplementaryData1.xlsx
- SupplementaryData2.xlsx
- SupplementaryData3.xlsx
- Supplementaryfiles.pdf

## Data Availability

The data generated during this study are provided in the main article and the supplementary files. Heavy and light chain sequences shown in Supplementary Data 1 have been deposited at GenBank with the accession codes PZ405537–PZ405758. The underlying primary datasets have been deposited in the Harvard Dataverse repository https://doi.org/10.7910/DVN/YEQIEH. The data are available through a formal request to the KEMRI-Wellcome Trust Research Programme Data Governance Committee if the use of the data is complaint with the consent provided by the participants. Details of the criteria can be found in the KEMRI-Wellcome data sharing guidelines (https://kemri-wellcome.org/data/). Requests for the data can be made to the Data Governance Committee (dgc@kemri-wellcome.org) through the corresponding authors.

## CODE AVAILABILITY

We have packaged the entire ‘*immbase*’ BCR precursor frequency pipeline with scripts and documentation as well as the scripts used for the novel allele analysis and haplotyping in a private repository that will become public upon manuscript publication https://github.com/ENduatiLab/IMMBASE_immune_repertoire_characterisation. TIgGER, Alakazam, Shazam and RAbHIT are tools from the Immcantation portal available here: https://immcantation.readthedocs.io/en/stable/index.html. The IgDiscover code used for participant IGHV genotyping is available from https://gitlab.com/gkhlab/igdiscover22.
